# A Triple Challenge: Cytomegalovirus and Ulcerative Colitis in a Human Immunodeficiency Virus-Positive Patient

**DOI:** 10.7759/cureus.78627

**Published:** 2025-02-06

**Authors:** Meumbur P Kpughur-Tule, Carly M Hubers, Ngumimi P Kpughur-Tule, Kendall Conway, Saba Asif, Alexander M Satei, Pritha Chitagi

**Affiliations:** 1 Internal Medicine, Trinity Health Oakland Hospital, Pontiac, USA; 2 Internal Medicine, Wayne State University School of Medicine, Detroit, USA; 3 Diagnostic Radiology, Wayne State University School of Medicine, Detroit, USA; 4 Diagnostic Radiology, Trinity Health Oakland Hospital, Pontiac, USA

**Keywords:** cmv colitis, coinfection, cytomegalovirus (cmv), human immunedeficiecy virus (hiv) infection, ulcerative colitis (uc)

## Abstract

Cytomegalovirus (CMV) colitis, ulcerative colitis (UC), and human immunodeficiency virus (HIV) infection represent distinct and complex conditions that pose significant diagnostic and therapeutic challenges, particularly when they occur concurrently. This case highlights a 44-year-old male with a history of UC and newly diagnosed HIV, presenting with refractory gastrointestinal symptoms ultimately attributed to CMV colitis superimposed on UC. Advanced diagnostic modalities, including endoscopic biopsy and polymerase chain reaction (PCR) testing, were instrumental in differentiating CMV colitis from a UC flare, guiding targeted antiviral and immunomodulatory therapies. This report notes the need for routine CMV screening in refractory UC, emphasizing the utility of quantitative PCR in identifying clinically significant infections. The case also highlights the intricate balance required in managing immune dysfunction, infection control, and inflammation in the context of HIV, CMV colitis, and UC. Furthermore, it draws attention to systemic barriers, such as medication access, which can exacerbate disease progression. Through a multidisciplinary approach, this case demonstrates the potential for tailored interventions to achieve favorable clinical outcomes and provides insights for managing similar complex conditions.

## Introduction

The co-occurrence of Cytomegalovirus (CMV) colitis and ulcerative colitis (UC) in patients newly diagnosed with Human immunodeficiency virus (HIV) is an exceedingly rare clinical phenomenon. Each condition alone poses significant diagnostic and therapeutic challenges, and their concurrence creates a complex interplay of immunological and gastrointestinal dysfunction. CMV is a ubiquitous herpesvirus that establishes lifelong latency in the host. While it remains asymptomatic in immunocompetent individuals, CMV can cause severe end-organ disease in immunocompromised patients, including those with HIV or post-transplant immunosuppression. CMV colitis, characterized by inflammation and ulceration of the colon, often presents with diarrhea, abdominal pain, and systemic symptoms such as fever [1].

HIV, the virus responsible for acquired immunodeficiency syndrome (AIDS), leads to progressive immune system deterioration by targeting CD4+ T lymphocytes. This immunosuppression predisposes individuals to opportunistic infections, including CMV, and complicates the management of autoimmune conditions [2]. UC, a chronic inflammatory bowel disease (IBD), involves recurring inflammation of the colon and rectum, resulting in symptoms such as diarrhea, rectal bleeding, and abdominal pain. The pathogenesis of UC is thought to involve a dysregulated immune response to intestinal microbiota in genetically predisposed individuals [3].

The intersection of these three conditions creates a complex clinical scenario. UC is primarily driven by dysregulated immune activation, often treated with immunosuppressive therapies. Conversely, HIV compromises immune function, frequently predisposing individuals to opportunistic infections such as CMV colitis. This dual pathology-immune suppression from HIV and the hyperactive inflammatory milieu of UC poses significant diagnostic and therapeutic dilemmas [4]. CMV remains latent in most individuals but can reactivate in those with compromised immune systems, such as patients with advanced HIV or AIDS. The depletion of CD4+ T cells in HIV weakens immune surveillance, increasing susceptibility to opportunistic infections like CMV colitis, which can mimic or exacerbate UC. Given this interplay, routine screening for CMV in immunocompromised patients with refractory gastrointestinal symptoms is essential to ensure timely diagnosis and appropriate management.

Diagnosing and managing these overlapping conditions require advanced diagnostic techniques and a multidisciplinary approach. Differentiating between a UC flare and CMV colitis often necessitates endoscopic biopsy and histopathological analysis. Moreover, therapeutic regimens can conflict, as CMV colitis necessitates antiviral therapy, while UC management often relies on immunosuppressive treatments [5]. This report highlights the diagnostic and therapeutic challenges in managing concurrent CMV colitis and UC in a patient newly diagnosed with HIV. By delving into this case, we aim to enrich the understanding of this rare intersection of diseases and its implications for clinical practice.

## Case presentation

A 44-year-old male with a known history of UC presented to the emergency department with four weeks of progressively worsening symptoms, including up to 20 episodes of bloody diarrhea daily, nausea, vomiting, significant weight loss of 35 pounds, and left lower quadrant abdominal pain. The patient reported profound fatigue and decreased oral intake due to nausea. He had been receiving Stelara (ustekinumab) for UC but had missed multiple doses over several months due to insurance barriers. His prior UC treatments included Humira (adalimumab), which was discontinued due to drug-induced psoriasis, and intermittent courses of prednisone. He had not followed up with his gastroenterologist for over a year.

On admission, the patient appeared chronically ill but was hemodynamically stable, with a heart rate of 88 beats per minute, blood pressure of 128/76 mmHg, and afebrile. Initial laboratory evaluation revealed leukocytosis (WBC 18.5 × 10⁹/L; reference range: 4.0-11.0 × 10⁹/L), elevated C-reactive protein (CRP 23.9 mg/L; reference range: <5.0 mg/L), and thrombocytosis (platelet count 671 × 10⁹/L; reference range: 150-400 × 10⁹/L). Hemoglobin was mildly reduced at 11.4 g/dL (reference range: 13.5-17.5 g/dL), consistent with chronic inflammation. 

**Figure 1:**
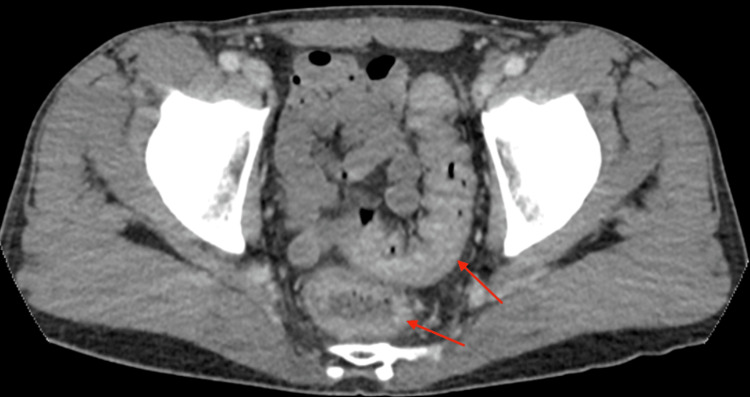
Axial CT imaging showing coloproctitis Axial reformat image of the abdomen and pelvis with IV contrast demonstrates mild wall thickening and enhancement involving the rectum and sigmoid colon, consistent with colitis.

**Figure 2:**
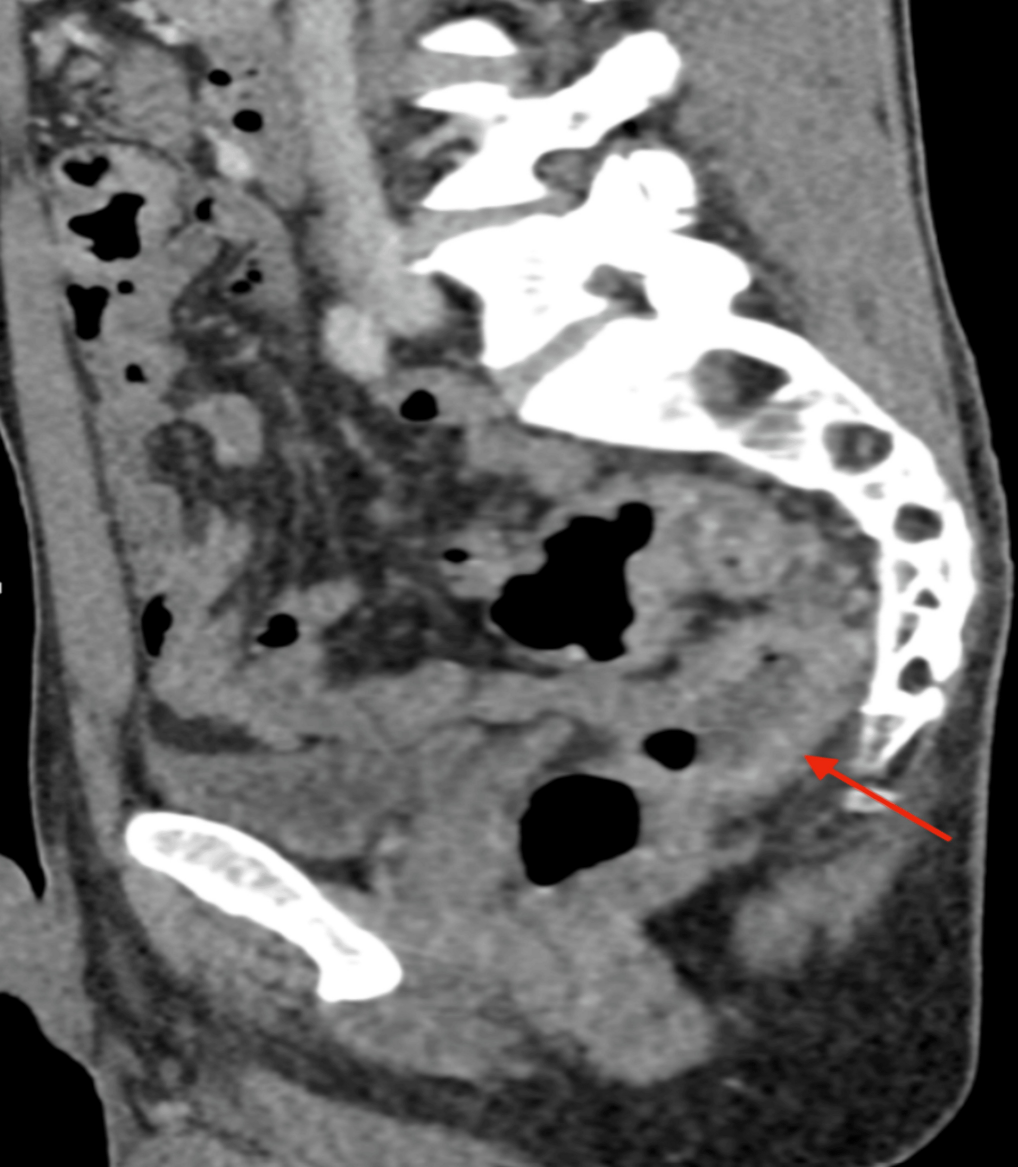
Sagittal CT imaging showing coloproctitis Sagittal reformat image of the abdomen and pelvis with IV contrast demonstrates mild wall thickening and enhancement involving the rectum and sigmoid colon, consistent with colitis.

Stool studies, including Clostridium difficile toxin and ova and parasite testing, were negative. CT imaging of the abdomen and pelvis demonstrated findings consistent with coloproctitis, including thickened and enhanced bowel walls, particularly involving the sigmoid colon (Figures 1-2).

The initial management of this patient included intravenous (IV) Solu-Medrol (methylprednisolone) at high doses, IV ceftriaxone, and IV metronidazole, in addition to supportive care with fluids, electrolyte repletion, and nutritional support. Despite these measures and receiving Stelara (ustekinumab) during hospitalization, the patient’s symptoms persisted with ongoing bloody diarrhea and abdominal pain.

**Figure 3:**
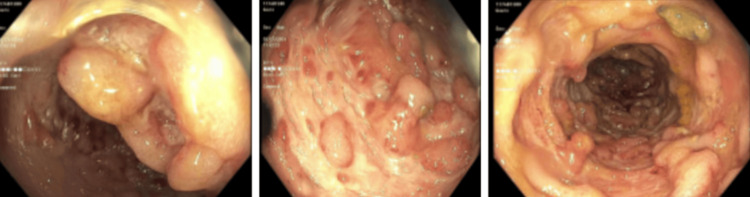
Sigmoidoscopy Sigmoidoscopy revealed severe sigmoid disease and inflammation characterized by erythema, granularity, mucus, pseudopolyps (left), inflamed mucosa (center), scarring and deep ulcerations (right) in a continuous and circumferential pattern. The rectum was spared.

Flexible sigmoidoscopy revealed severe sigmoid disease characterized by pseudopolyps, friable mucosa, and deep ulcerations sparing the rectum (Figure 3). The discrepancy between the colonoscopic finding of rectal sparing and the CT imaging suggestive of proctitis may be attributed to differences in disease distribution and timing of assessment. CT imaging can detect mucosal and submucosal edema, which may precede or persist beyond endoscopic mucosal changes. Histological examination confirmed severe inflammation consistent with UC. Due to his immunocompromised state and worsening clinical picture, further diagnostic workup was pursued. Serum CMV viral load was found to exceed 20,000 copies/mL (reference threshold for significance: >1,000 copies/mL), and HIV testing revealed newly diagnosed HIV with a CD4 count of 256 cells/μL (reference range: 500-1,500 cells/μL). A summary of all laboratory findings can be found in Table 1. Notably, HIV screening was prompted by the combination of severe immunosuppression and refractory gastrointestinal symptoms.

**Table 1:** Laboratory Findings in a 44-Year-Old Male with Concurrent CMV Colitis, Ulcerative Colitis, and HIV Infection This table summarizes the key laboratory findings observed in the patient. The results highlight significant leukocytosis, thrombocytosis, and anemia, along with elevated inflammatory markers such as C-reactive protein (CRP). The elevated serum CMV viral load and reduced CD4 count indicate active CMV infection and immunosuppression, respectively. Normal ranges are included for comparison.

Parameter	Patient Value	Reference Range	Interpretation
White Blood Cell (WBC) Count	18.5 × 10⁹/L	4.0-11.0 × 10⁹/L	Elevated (Leukocytosis)
Platelet Count	671 × 10⁹/L	150-400 × 10⁹/L	Elevated (Thrombocytosis)
Hemoglobin (Hb)	11.4 g/dL	13.5-17.5 g/dL	Low (Mild Anemia)
C-Reactive Protein (CRP)	23.9 mg/L	<5.0 mg/L	Elevated (Inflammatory Response)
Serum CMV Viral Load	>20,000 copies/mL	>1,000 copies/mL threshold	Significantly Elevated (Active CMV)
CD4 Count	256 cells/μL	500-1,500 cells/μL	Low (Immunosuppressed)

Given the CMV colitis diagnosis, infectious disease consultation was sought. The patient was promptly initiated on IV ganciclovir, followed by a gradual transition to oral valacyclovir upon clinical improvement. Concurrently, antiretroviral therapy (ART) with Biktarvy (bictegravir, emtricitabine, and tenofovir alafenamide) was initiated. To manage his UC, the patient was transitioned to Rinvoq (upadacitinib), a selective JAK inhibitor, due to persistent symptoms despite prior Stelara (ustekinumab) use.

Over his 28-day hospital course, the patient’s symptoms gradually improved, with bowel movements decreasing to four non-bloody episodes daily. His abdominal pain resolved, and he reported improved appetite and energy levels. By the time of discharge, he was tolerating a regular diet. He was discharged on a regimen of oral valacyclovir, prednisone taper, Biktarvy (bictegravir, emtricitabine, and tenofovir alafenamide), and scheduled follow-ups with gastroenterology, infectious disease, and colorectal surgery. A detailed care plan was provided to address medication adherence, follow-up imaging, and laboratory monitoring to assess treatment response and detect potential complications.

## Discussion

The interplay of CMV colitis, UC, and HIV presents a complex diagnostic and therapeutic challenge, requiring nuanced consideration of their pathophysiological interactions. CMV colitis, while rare, should always be considered in the differential diagnosis for patients with UC who present with refractory symptoms, particularly in the context of immunosuppression. HIV and CMV co-infection occurs primarily due to the immunosuppressive effects of HIV, which weaken the host’s ability to control latent infections. While these viruses differ in their genetic composition-HIV being an RNA virus and CMV a DNA virus-they coexist within the same individual because HIV-induced CD4+ T cell depletion impairs immune surveillance, allowing CMV to reactivate. CMV remains latent in monocytes, endothelial cells, and tissue macrophages, while HIV actively replicates in CD4+ T cells. In immunocompromised individuals, particularly those with HIV/AIDS, CMV reactivation is common and can lead to end-organ disease, including colitis. This highlights the importance of considering CMV infection in HIV-positive patients presenting with gastrointestinal symptoms, as undiagnosed CMV colitis can contribute to significant morbidity.

This case is not isolated; similar reports have demonstrated that CMV infection can mimic or exacerbate UC flares. For instance, a case by Khan et al. described a case where CMV colitis presented with symptoms that were very similar to a UC exacerbation, such as abdominal pain, diarrhea, and mucosal ulcerations. As demonstrated in this report, misattributing these symptoms to UC alone can delay appropriate treatment. The study emphasizes the importance of histopathological evaluation, including the identification of hallmark features such as "Owl’s-Eye" intranuclear inclusions, to confirm CMV colitis [6]. Early recognition of CMV in such scenarios is critical to prevent severe complications such as colonic perforation or toxic megacolon.

Histopathological confirmation remains the gold standard for diagnosing CMV colitis. In our patient, sigmoidoscopy revealed severe sigmoid disease with pseudopolyps and deep ulcerations, consistent with CMV colitis superimposed on UC. This calls attention to the importance of biopsy in confirming the diagnosis and the complementary role of CMV polymerase chain reaction (PCR) testing in distinguishing CMV colitis from a UC flare. A study by Wethkamp et al. further highlighted the critical value of routine CMV screening in refractory UC, demonstrating that patients treated with antiviral therapy achieved significantly better outcomes. The study emphasized the pivotal role of quantitative real-time PCR testing, which offers superior sensitivity compared to traditional methods like immunohistochemistry or histological evaluation, particularly for detecting CMV at lower viral loads. By establishing a viral load threshold of 600 CMV copies per 10^5 cells, the study provided a reliable marker for identifying clinically relevant infections requiring antiviral treatment [7]. Moreover, the ability to perform PCR testing on formalin-fixed, paraffin-embedded biopsy samples enhances its practicality, addressing logistical challenges associated with fresh tissue samples. This precision in detecting clinically significant infections allows for better patient stratification and tailored antiviral therapy, ultimately leading to improved outcomes in refractory UC cases.

A meta-analysis by Qin et al. provides critical insights into the risk factors for CMV reactivation in patients with UC. Severe UC and pancolitis emerged as significant predictors of CMV reactivation, with odds ratios of 1.465 and 2.108, respectively, indicating a higher likelihood of reactivation in these conditions due to intense inflammatory responses and compromised mucosal barriers [8]. Furthermore, the study identified glucocorticoids and immunosuppressive agents, including azathioprine, as strong contributors to CMV reactivation, emphasizing the immunomodulatory burden of these therapies. These findings underscore the necessity for proactive CMV screening in patients with severe UC or those receiving immunosuppressive treatments to enable timely antiviral intervention and improve clinical outcomes​

Management of these overlapping conditions involves balancing immunosuppression and infection control. Ganciclovir, an antiviral with potent activity against CMV, is the first-line treatment for CMV colitis. However, its immunosuppressive effects can complicate UC management. In our case, the patient’s clinical improvement with ganciclovir highlights its efficacy but also underscores the importance of monitoring for the reactivation of UC symptoms. Similar cases in the literature have reported success with combining antiviral therapy with a tailored approach to UC management, often involving steroid-sparing agents or biologics like tofacitinib [5].

The initiation of ART in this patient added another layer of complexity. While ART restores immune function and prevents further opportunistic infections, it carries the risk of immune reconstitution inflammatory syndrome (IRIS). IRIS can unmask subclinical infections or worsen underlying inflammatory conditions, necessitating close monitoring [9]. In our patient, ART was initiated concurrently with antiviral therapy and immunomodulation for UC, with no signs of IRIS during follow-up. This suggests that multidisciplinary coordination and careful timing of interventions can mitigate risks.

Systemic barriers, such as medication access, also played a significant role in this case. Delayed Stelara administration due to insurance issues contributed to disease progression and highlighted the need for healthcare systems to prioritize accessibility for patients with chronic conditions. Addressing these systemic issues could prevent similar cases of exacerbated disease and reduce the burden on healthcare systems.

The management of overlapping diseases such as CMV colitis, ulcerative colitis, and HIV in resource-limited settings highlights the importance of accessibility to effective treatments and healthcare interventions. Lessons can be drawn from schistosomiasis control programs, which have successfully implemented large-scale preventive chemotherapy and public health initiatives to reduce disease morbidity. Such programs emphasize the necessity of coordinated efforts combining medical treatment, access to essential drugs, and public health measures like sanitation and health education to address complex, multifactorial diseases. Similarly, a comprehensive approach integrating antiviral therapies, immune modulation, and support systems for medication adherence can mitigate the compounded burden of CMV colitis and UC in immunocompromised individuals, ultimately reducing long-term complications and healthcare costs [10].

## Conclusions

In conclusion, the co-occurrence of CMV colitis and UC in an HIV-positive patient represents a rare and complex clinical challenge requiring precise diagnostic and therapeutic strategies. This case highlights the importance of considering opportunistic infections like CMV in patients with refractory UC, particularly those with underlying immunodeficiency. Our patient demonstrated significant immunosuppression, evidenced by a low CD4+ T cell count of 256 cells/μL, alongside an elevated CMV viral load exceeding 20,000 copies/mL. These findings underscore the role of HIV-induced immune dysfunction in facilitating CMV reactivation and colitis development. Early recognition and targeted treatment, including antiviral therapy for CMV, immunomodulatory therapy for UC, and antiretroviral therapy for HIV, were critical in achieving clinical improvement. Multidisciplinary collaboration remains pivotal in navigating such complex cases, ensuring optimal patient outcomes, and guiding future management of similar presentations.
